# Notes and News

**Published:** 1905-08-19

**Authors:** 


### Notes and News.

A new operating theatre has been opened at the North Charitable Infirmary, Cork. It was presented by Miss Honan.

The Governors of the Manchester Children's Hospital have received a legacy of £12,000 from the executors of the late Mr. Godfrey Ermen. It will be devoted to a new dispensary and out-patients' department.

At a meeting held at Devizes last week Dr. Weatherley made an offer of a bed at the Winsley Consumption Sanatorium to Devizes for £250. The offer is open to consideration for one month. The cost of maintenance at Winsley is under 25s. per week.

The Committee of the Bristol Royal Infirmary passed a vote of thanks to Mr. Samuel White for his efforts on behalf of the institution, and especially for those which he devoted to the success of the Bristol Carnival, and they resolved to name a ward the "Samuel White Ward" in recognition of his services.

A kindly thought prompted those who arranged the luncheon given by the Houses of Parliament to the French Fleet officers to forward the fruit which was not consumed to the Hospital for Sick Children, Great Ormond Street. The fruit was mostly provided for the occasion by members of Parliament. The hospital has also recently received from the Commissioner for Trinidad a gift of Trinidad bananas.

Two interesting books are now in the press. The first is a work by C. J. Lewis, M.D., D.Sc., a well-known authority on public bealth, and Mr. J. N. Lewis, Fellow of the Institute of Actuaries, analysing the Scottish births registered in the first year of registration. In this work statistics will be comprehensively dealt with, and interesting deductions drawn from the results. The other book in course of publication by Messrs. Oliver and Boyd is "Scottish Sanitary Laws and Principles of Public Health," by Dr. W. Broek, Medical Officer of Health for Midlothian.

The Hospital Sunday Fund collection this year has already reached the sum of £73,780.

Major C. Spencer, F.R.C.S.E., R.A.M.C., has been appointed Professor of Military Surgery at the Royal Army Medical College, which has become vacant by the retirement of Surgeon-General W. Stevenson, C.B.

The late Miss Mary Chapman, of Carlisle Place, has bequeathed £500 each to the Cancer Hospital and to the Victoria Hospital for Children. The late Mr. Thomas John Bell has bequeathed £5,000 to King Edward's Hospital Fund.

The interesting series of engraved mezzotint portraits of famous physicians, surgeons, and medical writers, formed early in the last century by the well-known antiquarian, T. J. Pettigrew, formerly librarian to the Duke of Sussex, will be sold by Messrs. Knight, Frank and Rutley at their rooms in Conduit Street on the 23rd inst.

Mr. L. C. McCausland has been appointed secretary to the Queen's Jubilee Hospital. Mr. McCausland has been promoted from the office of steward at Westminster Hospital, and we congratulate the Queen's Jubilee Hospital on securing as their secretary one who has had previous hospital experience. He will take up his duties early next month.

The outbreak of yellow fever in New Orleans is proving exceedingly severe, and the disease has appeared in the district; over 1,000 cases are recorded. No shipments of bananas are now permitted from the city. Professors Boyce and Ross, of the Liverpool School of Tropical Medicine, are proceeding to New Orleans to offer their services to the authorities.

An interesting feature in connection with the Sailors' Palace, the Home and Institute now being erected in Copenhagen, is the Nelson Centenary Home, which is to be equipped by friends in this country, for the use of British seamen when in the port of Copenhagen. The Queen has already contributed £10 to the scheme, through the British and Foreign Sailors' Society, Passmore Edwards Sailors' Palace, Limehouse, E. The society will be glad to receive contributions.

Sir Edmund Hay Currie, the secretary of the Hospital Sunday Fund, has forwarded to the secretaries of the 80 hospitals in the metropolis having out-patient departments the following resolution of the distribution committee:—"This committee is of opinion that the serious increase in the number of out-patients (many of whom have trivial complaints) is detrimental to the welfare of hospital and patients, inasmuch as they are a burden to the funds of the hospitals and prevent the hospital staff from giving the necessary attention to serious cases." The committees of the hospitals are invited to express their opinions on this subject and, if they agree, to suggest a remedy, before the end of the present financial year.

